# Comparison of Kinematics in Cruciate Retaining and Posterior Stabilized for Fixed and Rotating Platform Mobile-Bearing Total Knee Arthroplasty with respect to Different Posterior Tibial Slope

**DOI:** 10.1155/2018/5139074

**Published:** 2018-06-11

**Authors:** Kyoung-Tak Kang, Yong-Gon Koh, Juhyun Son, Oh-Ryong Kwon, Jun-Sang Lee, Sae Kwang Kwon

**Affiliations:** ^1^Department of Mechanical Engineering, Yonsei University, 50 Yonsei-ro, Seodaemun-gu, Seoul 03722, Republic of Korea; ^2^Joint Reconstruction Center, Department of Orthopaedic Surgery, Yonsei Sarang Hospital, 10 Hyoryeong-ro, Seocho-gu, Seoul 06698, Republic of Korea

## Abstract

Reconstructed posterior tibial slope (PTS) plays a significant role in kinematics restoration after total knee arthroplasty (TKA). However, the effect of increased and decreased PTS on prosthetic type and design has not yet been investigated. We used a finite element model, validated using in vitro data, to evaluate the effect of PTS on knee kinematics in cruciate-retaining (CR) and posterior-stabilized (PS) fixed TKA and rotating platform mobile-bearing TKA. Anterior-posterior tibial translation and internal-external tibial rotation were investigated for PTS ranging from -3° to 15°, with increments of 1°, for three different designs of TKA. Tibial posterior translation and external rotation increased as the PTS increased in both CR and PS TKAs. In addition, there was no remarkable difference in external rotation between CR and PS TKAs. However, for the mobile-bearing TKA, PTS had less effect on the kinematics. Based on our computational simulation, PTS is the critical factor that influences kinematics in TKA, especially in the CR TKA. Therefore, the surgeon should be careful in choosing the PTS in CR TKAs.

## 1. Introduction

Total knee arthroplasty (TKA) is one of the most successful orthopaedic surgical treatments to eliminate pain and improve knee function in patients with knee osteoarthritis (OA), showing excellent long-term survivorship [1, 2]. The goal of TKA is to restore normal knee function and kinematics. To achieve this, the reconstructed tibiofemoral (TF) joint should be stable in the full range of knee flexion.

The factors that affect the kinematics are influenced by the patient's condition, the surgical technique, and the implant design [3]. Among these factors, it has been reported that increased posterior tibial slope (PTS) is related to increased postoperative range of motion (ROM) in cruciate-retaining (CR) TKA, because of more consistent femoral rollback and reduced impingement of the polyethylene (PE)insert against the posterior femur [4–7]. In addition, some cadaver studies have evaluated the relationship between the PTS and kinematics in CR TKA [8, 9]. In contrast, there have been few reports concerning the PTS in posterior-stabilized (PS) TKA [10]. Further, whether increased PTS is clinically advantageous or not is still a controversial topic [11–13].

Design evolution and variability have a direct influence on the kinematics in knee joints, including kinematic stability [14, 15]. Both CR and PS TKA offer surgeons a wide variety of selection of PE inserts for varying conformity, shape, slope, design, and femoral morphology for single- or multiradius designs and symmetric or asymmetric femoral condyles [16]. All of these factors influence kinematic stability [17, 18]. In contrast to a fixed-bearing TKA, a mobile-bearing TKA includes a rotating platform that provides higher conformity of prosthetic components to articular surfaces. It may produce reproducible anteroposterior (AP) translation between the components during daily activities [19, 20].

Numerous kinematic analyses have been conducted previously with respect to different prosthetic designs; fixed-bearing CR and PS TKA, and rotating platform mobile-bearing TKA [4–6, 14, 16, 18, 20–22]. However, there have been several discrepancies in these studies [4–6, 14, 16, 18, 20]. Potential reasons for these differences may include different TKA implant manufacturers and designs, confounding variables such as in vivo soft tissue contractures that are difficult to control in vitro, and sample size. Further, to the best of our knowledge, there have been no studies conducted to evaluate the kinematics for different prosthetic designs, with respect to variations in the PTS. In the present study, the advantage of using a computational simulation that uses a single subject is that the effects of the component alignment within the same subject could be determined without being affected by intersubject variability such as weight, height, bone geometry, ligament properties, and component size [23].

The purpose of this study was to determine the kinematic changes for fixed-bearing CR and PS TKA and rotating platform mobile-bearing TKA using a validated finite element (FE) model. We evaluated AP translation and internal-external (IE) rotation in the TF joint under the deep knee bend condition. We hypothesized that the PTS was the most significant factor influencing the kinematics in CR TKA.

## 2. Materials and Methods

The model used in this study included features based on a validated knee joint FE model presented in a previous study [24–27]. A three-dimensional (3D) nonlinear FE model of a normal knee joint was developed using data from computed tomography (CT) and magnetic resonance imaging (MRI) scans of a healthy 37-year-old male subject. The CT and MRI models were developed with slice thicknesses of 0.1 mm and 0.4 mm, respectively. The medical history of the subject revealed no musculoskeletal disorders or related diseases arising from a malalignment in the lower extremity, thereby indicating a healthy knee joint.

The reconstructed CT and MRI models were combined with positional alignment of each model by using commercial software, Rapidform (Version 2006; 3D Systems Korea Inc., Seoul, South Korea), to model the bone structures as rigid bodies using four-node shell elements [28]. The major ligaments were modeled using nonlinear and tension-only spring elements [29, 30]. The ligament insertion points were chosen considering the anatomy obtained from the MRI sets of the subject and the descriptions based on previous studies [31–33]. Two experienced orthopaedic surgeons determined the locations of the ligaments independently. The agreement was evaluated using the 3D coordinates of each point. Intraclass correlation coefficients for intra- and interrater agreement ranged from 0.86 to 0.96 for all measurements, which showed good reproducibility.

**Figure 1:**
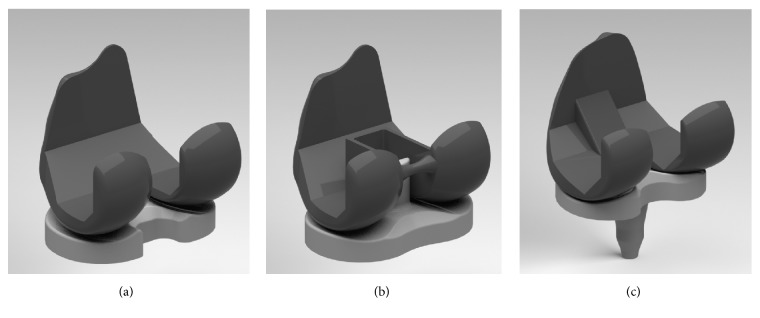
Schematics of (a) CR fixed-bearing, (b) PS fixed-bearing, and (c) rotating platform mobile-bearing TKA.

To develop the changed PTS models, surgical simulation of a TKA was performed by two experienced surgeons. Computer-assisted design models of both CR and PS fixed-bearing designs from the Genesis II Total Knee System (Smith & Nephew, Inc., Memphis, TN, USA) and rotating platform mobile-bearing design from LCS (Johnson & Johnson/ DePuy, Warsaw, IN, USA) were virtually implanted into the bone geometry (Figure 1). Femoral component size 7 and tibial insert size 5-6 were selected for fixed-bearing TKA, and femoral component size large and tibial baseplate size 4 were selected for mobile-bearing TKA, based on the dimensions of the femur and the tibia, respectively.

**Figure 2:**
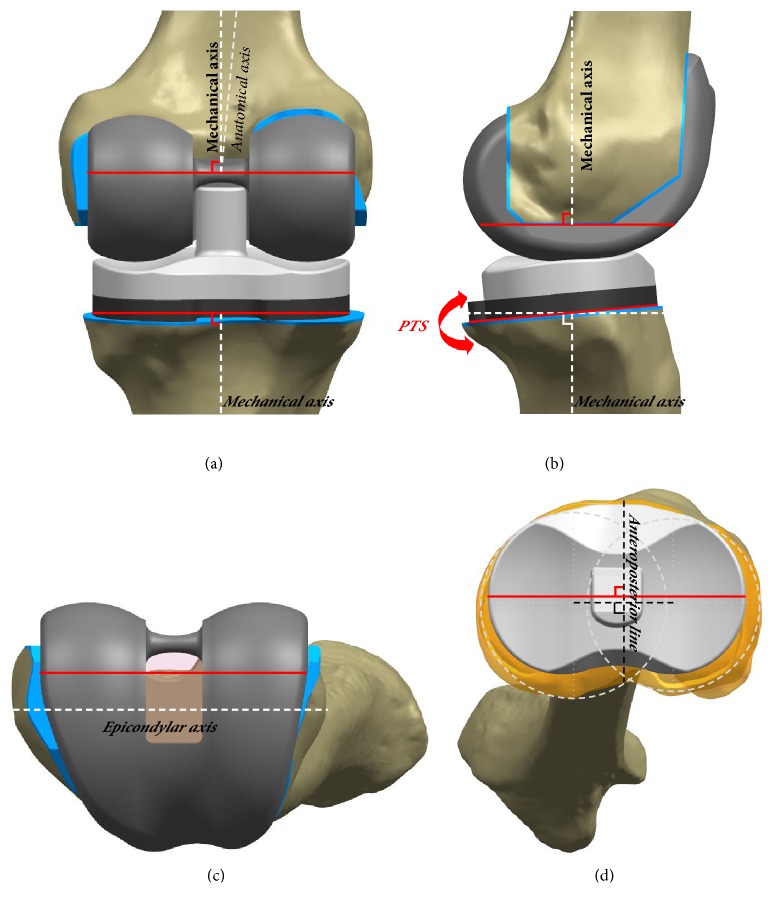
The orientation of the TKA used in this study in the (a) coronal plane, (b) sagittal plane, and (c, d) transverse plane.

In the neutral position, in aligning the components in the coronal plane, the femoral component was set perpendicular to the mechanical axis connecting the center of the knee and the center of the femoral head. The tibial component was set perpendicular to the mechanical axis connecting the center of the knee and the center of the ankle joint [34]. The rotational alignments of the femoral and tibial components were positioned in line with the femoral epicondylar and tibial anteroposterior axes, respectively (Figure 2). Tibial rotational alignment is the anteroposterior line bisecting a line connecting the circle centers perpendicularly based on Cobb's et al. study [35].

**Figure 3:**
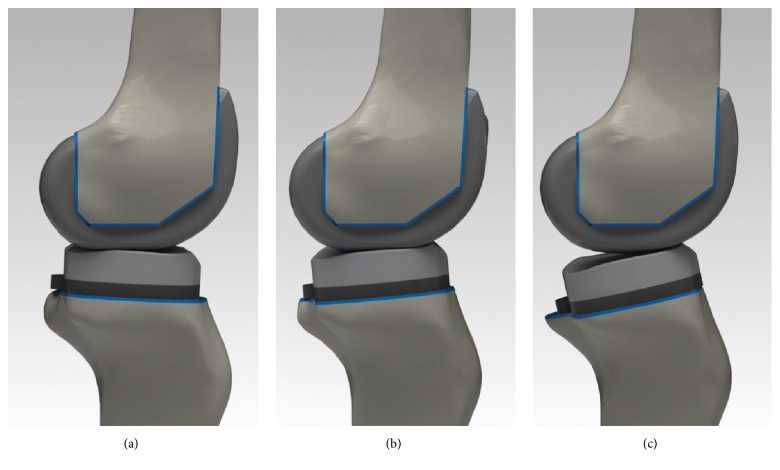
FE models used in this study of CR fixed-bearing TKA with respect to different PTS: (a) -3°; (b) 6°; and (c) 15°.

In order to facilitate changing of the PTS, the pivot point was defined as the midpoint between the centers of the medial and lateral tibial plateaus (Figure 2(b)). In this condition, a more PTS would shift distally all the points located posteriorly to the pivot point, and proximally all the points located anteriorly, and vice versa for a more anterior slope (Figure 3) [36]. Nineteen FE models for each prosthetic design (57 FE models in total) were developed from -3° PTS to 15° PTS, with increments of 1°. The range of PTS angle was determined from previous studies [37, 38].

This corresponds to the lowest point of the PE insert articular surface adjacent to the lowest points of the femoral articular surfaces in extension. Contact conditions were applied to the femoral component, PE insert, and patellar button in TKA. The coefficient of friction between the PE material and metal was selected as 0.04, for consistency with the explicit FE models proposed in previous studies [39]. The femoral component, PE insert, tibial component, and bone cement were made of cobalt-chromium-molybdenum (CoCrMo) alloy, ultra-high-molecular-weight-polyethylene (UHMWPE), titanium (Ti6Al4V) alloy, and poly (methyl methacrylate) (PMMA), respectively. In a manner similar to that in previous studies, the materials were assumed to be homogeneous and isotropic, except for the PE insert (Table 1) [39–43], which was modeled as an elastoplastic material. The UHMWPE had a yield strength and ultimate tensile stress of 17 MPa and 33 MPa, respectively [39]. The cement layer was considered with a constant penetration depth of 3 mm into the bone, based on a test for different cementing techniques at the femoral and tibial resection surfaces in contact with the femoral and tibial components, respectively [44, 45]. The interfaces between the prosthesis and the bone were rigidly fixed by the cement used [42, 46].

The PTS change model topologies provided six degrees of freedom to the TF and PF joints. Under the first loading condition, 150 N was applied to the tibia with 30° and 90° flexion in the FE knee joint in order to measure the anterior tibial translation and posterior tibial translation [47]. Additionally, a second axial loading of 1,150 N was applied to the model in order to obtain the contact stresses and compare them to those reported by a published FE study on the knee joint [28]. Under the third to fourth loading condition, the TKA model was validated by comparing it to the models used in previous studies [48–50]. A conservative ankle force of 50 N and a hamstring force of 10 N were continuously exerted in a linearly increasing manner [51], to a maximum of approximately 600 N at a 90° flexion angle of the quadricep actuators, for the fixed-bearing TKA model under the first loading condition [48, 49]. In addition, 133 N anterior forces and 89 N posterior forces were applied in 30° and 75° flexions, followed by measurement of the total AP displacement, to validate the mobile-bearing TKA model under the fourth loading condition [50]. The fifth loading condition corresponded to the deep knee bend loading, applied to evaluate the effects of the increased PTS. A computational analysis was conducted with an AP force applied to the femur, corresponding to the compressive load applied to the hip [52–54]. A proportional-integral–derivative controller was incorporated into the computational model to control the quadriceps in a manner similar to that in a previous experiment [55]. A control system was used to calculate the instantaneous displacement of the quadriceps required to match the target flexion profile, which was the same as that in the aforementioned experiment [55]. IE and varus-valgus torques were both applied to the tibia [52–54].

The FE model was analyzed using the ABAQUS software (Version 6.11; Simulia, Providence, RI, USA). The movement of the contact point and the kinematics in the TF joint were calculated throughout the deep-knee-bending task. The contact point was calculated based on the motion of the center of contact stress. The kinematics was calculated based on Grood and Suntay's definition of a joint coordinate system [56].

## 3. Results

### 3.1. Validation of the Intact and TKA Model

For validation purposes, the intact FE model was compared to the experiment with its own subject. Under the loading condition with a 30° flexion, the anterior tibial translation was 2.83 mm in the experiment and 2.54 mm in the FE model. The posterior tibial translation was 2.12 mm in the experiment and 2.18 mm in the FE model. Similarly, with 90° flexion, the anterior tibial translation was 3.32 mm in the experiment and 3.09 mm in the FE model. The posterior tibial translation was 2.64 mm in the experiment and 2.71 mm in the FE model, which showed a good agreement with those obtained by the FE model [47].

Additionally, the model was validated by comparison with experiments obtained by previous studies. Average contact stresses of 3.1 MPa and 1.53 MPa were observed on the medial and lateral meniscus, respectively, under an axial load of 1,150 N. Both were within 6% of the 2.9 MPa and 1.45 MPa contact pressure values reported by Peña et al. [28]. These minor differences could have been caused by geometrical variations between the different studies, such as the thickness of the cartilage and meniscus. Overall, however, the considerable consistency between the validation results and the results reported in the literature confirmed the validity of the results obtained by the FE model used in this study.

**Figure 4:**
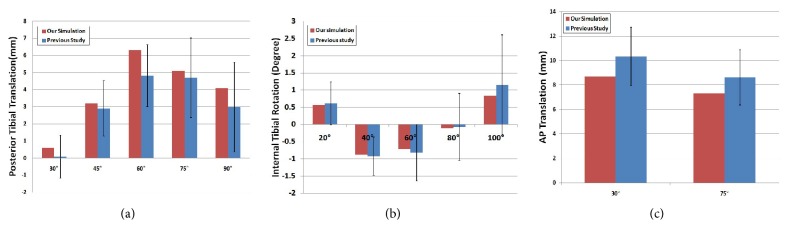
The validation with comparison between FE analysis and previous experimental studies for three different types of prostheses: (a) posterior tibial translations in CR TKA; (b) internal tibial rotations in PS TKA; and (c) AP translations in mobile-bearing TKA.

Moreover, the kinematics was compared to the experimental results obtained by previous studies in order to validate the TKA FE model. Posterior tibial translations in the CR TKA model were 0.6 mm, 3.2 mm, 6.3 mm, 5.1 mm, and 4.1 mm, for 30°, 45°, 60°, 75°, and 90° flexions (Figure 4(a)), respectively [49], and the internal tibial rotations in the PS TKA model were 0.57°, -0.88°, -0.71°, -0.11°, and 0.83°, for 20°, 40°, 60°, 80°, and 100° flexions (Figure 4(b)), respectively [48]. For mobile-bearing TKA, AP translations in TF joint were 8.7 mm and 7.3 mm in 30° and 75° flexions (Figure 4(c)), respectively [50]. These simulation results showed good agreement with previous experimental studies within the ranges of values under identical loading conditions, as applied to the prosthetic implant [48–50].

### 3.2. TF Kinematics and Contact Point with respect to the PTS Change in Fixed-Bearing CR and PS TKA and Mobile-Bearing TKA

**Figure 5:**
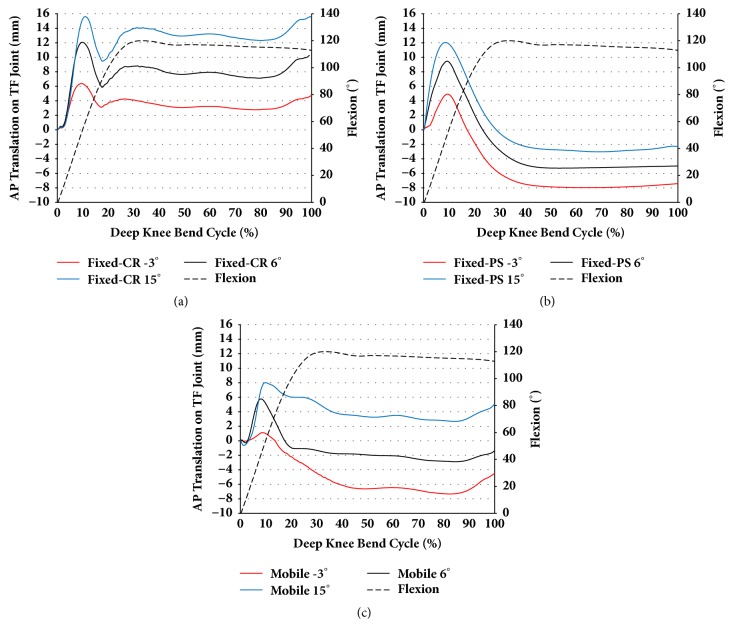
The comparison of AP translation with respect to different PTS for three types of prostheses during deep knee bend simulation: (a) CR TKA; (b) PS TKA; and (c) mobile-bearing TKA.

**Figure 6:**
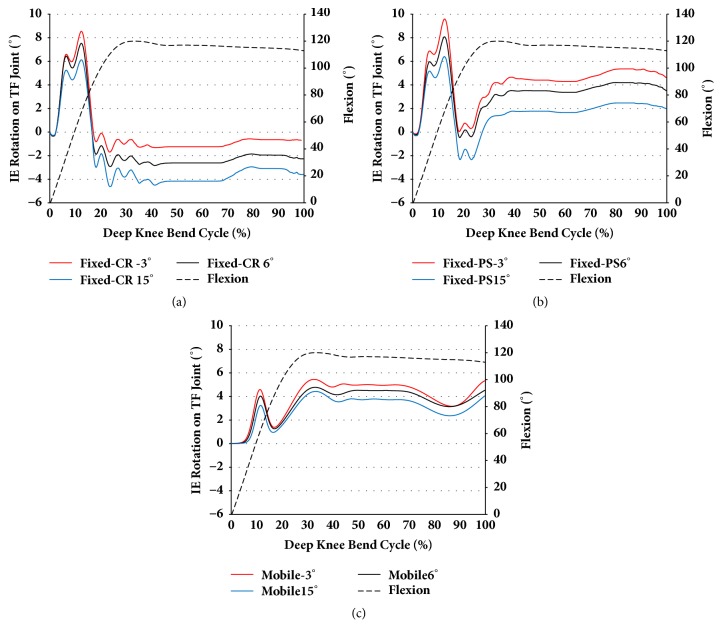
The comparison of IE rotation with respect to different PTS for three types of prostheses during deep knee bend simulation: (a) CR TKA; (b) PS TKA; and (c) mobile-bearing TKA.

Figures 5 and 6 show the AP translation and IE rotation for the TF joint with respect to the change in the PTS under the deep knee bend condition in fixed-bearing CR and PS and mobile-bearing TKA. This trend was most significantly observed in fixed-bearing CR TKA, followed by fixed-bearing PS TKA and mobile-bearing rotating platform TKA. The amplitude of AP translation increased as the PTS increased in both fixed-bearing CR and PS TKA. However, in mobile-bearing rotating platform TKA, there was no difference in amplitude of AP translation with an increase in PTS. Amplitude increased by 30% and 27% in fixed-bearing CR and PS TKA for PTS 15° compared to 6° model. However, in mobile-bearing rotating platform, amplitude was constant.

TF joint external rotations increased as the PTS increased in fixed-bearing CR and PS TKA. However, in contrast to AP translation, IE rotation was not influenced by change in the PTS. Further, the amplitude of IE rotation was not influenced by PTS change in both CR and PS TKA. In addition, internal rotation of the TF joint increased as PTS increased in mobile-bearing rotating platform TKA.

**Figure 7:**
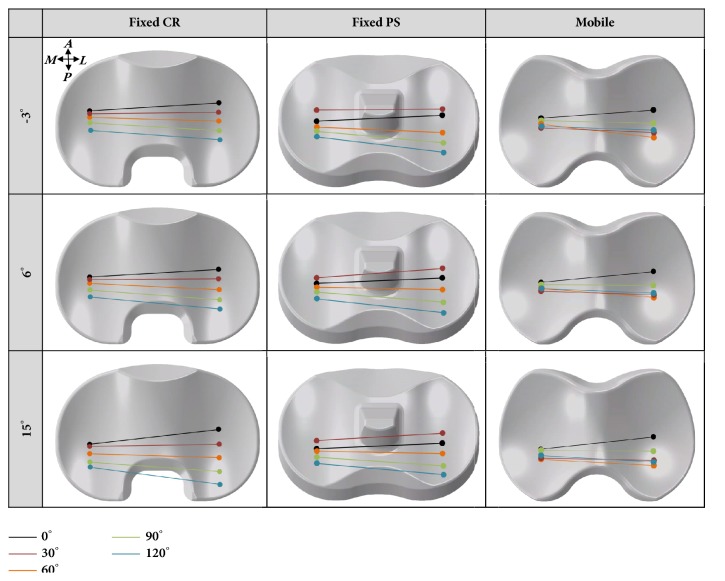
The comparison of change in contact point with respect to different PTS for three types of prostheses during deep knee bend simulation.

Figure 7 shows the change in the contact point in fixed-bearing PS and CR TKA and mobile-bearing rotating platform TKA. The overall posterior locations of the TF contact points were determined to be in the medial and lateral compartments across all motor tasks as the PTS increased for all three prosthetic designs. Additionally, the lateral contact point in the TF joint translated in the posterior direction with an increase in the flexion, relative to medial contact point, irrespective of the increase in the PTS for all three prosthetic designs. The distance travelled by the TF joint contact point increased on both the medial and lateral side, with a higher PTS in both fixed-bearing CR and PS TKA. Based on the motion of the center of pressure in the TF joint, the AP translation in mobile-bearing rotating platform TKA was not sensitive to the motion of the center of pressure in the TF joint

## 4. Discussion

Posterior TF translation is important in TKA due to the prior TF impingement that might occur during flexion [57]. Previous study showed that posterior TF translation significantly increased by 3.1 mm as PTS increased by 3° each in fixed-bearing CR TKA [58]. Such a trend is similar to our result, but there is slight difference. Potential reasons for this difference may include different TKA implant manufacturers/designs, confounding variables such as soft tissue contractures and posterior cruciate ligament (PCL) integrity that are difficult to control in vitro, and sample size. Previous study showed that anterior impingement between the anterior aspect of the tibial post and the femoral component occurred as PTS increased in fixed-bearing PS TKA [38]. However, there is no anterior impingement in our study. The main reason was that cutting was made with the center of tibial plateau in PTS model, not an anterior tibial cortex. In addition, this is the first study to our knowledge that has shown a correlation between PTS and posterior TF translation in fixed-bearing CR and PS and mobile-bearing TKA.

The most important finding in this study was that PTS was the critical factor that influences postoperative kinematics in TKA. The primary outcome of our study is evidence of comparable kinematic outcomes for fixed-bearing CR and PS TKA and mobile-bearing rotating platform TKA, confirmed by evaluation of AP translation and IE rotation under deep knee bend conditions.

The knee joint kinematics under the deep keen bend condition have not been previously studied after implantation of fixed-bearing CR or PS TKA and mobile-bearing rotating platform TKA, with respect to differing PTSs. A previous study reported that muscle forces are important to create knee translation and rotation [59]. Because previous studies have suggested that TF translation and rotation are important in achieving maximal flexion due to the rollback phenomenon, we feel that achieving flexion via application of a force to the native knee flexor muscles (hamstrings) may allow for a more physiological method to assess TF joints translated in the posterior direction and under flexion [57, 60]. The translation of TF joints in the posterior direction significantly increased by 3.2 mm, 2.3 mm, and 1.1 mm in fixed-bearing CR and PS TKA and mobile-bearing rotating platform TKA, respectively, during the same interval increase in flexion of PTS from 1° to 4°. To the best of our knowledge, this is the first study that has evaluated a correlation between the PTS and TF joints translated in fixed-bearing CR and PS TKA and mobile-bearing rotating platform TKA. As previously mentioned, posterior TF translation is important in TKA, because it allows more flexion prior to TF impingement [57]. In addition, a more posterior TF contact point at full flexion improves the moment arm of the quadriceps and has been associated with improvement in International Knee Society Function scores for CR TKA [61, 62]. However, unlike CR TKA, the post-cam mechanism of PS TKA can theoretically prevent an anterior femoral translation in flexion, leading to posterior impingement, even with decreased PTS. Our result also showed that CR TKA was most influenced by change in PTS in TF joint translation.

Theoretically, restoration of normal PCL function and PTS in CR TKA can restore normal knee kinematics. However, the surgical procedure does not always accomplish this, causing nonphysiological knee kinematics and geometry. In these cases, excessive PCL tension or posterior impingement associated with PCL dysfunction can limit postoperative flexion in CR TKA [63]. However, in the present study, the advantage of the computational simulation using a single subject was to allow evaluation of the correlation in controlled TF joint translation with respect to differing PTS.

The fixed-bearing and mobile-bearing TKA demonstrated a relatively asymmetrical posterior femoral translation during flexion in the contact point analysis. This is less than the maximum amount of posterior femoral rollback in the normal knee [64]. Although there was no significant difference in the amount of posterior femoral rollback between the two different prosthetic designs, the pattern of the medial and lateral condyle motion was different. In mobile-bearing TKA, the medial condyle experienced anterior motion, followed by posterior translation with progressive knee flexion, and the lateral condyle translated posteriorly during the process of flexion, the anterior motion of medial condyle, and posterior translation of lateral condyle showed central pivot rotation. This is similar to previously reported results [65, 66]. In a normal knee, the tibia always experiences medial pivot internal rotation during knee flexion [64]. We found that fixed-bearing CR and PS TKA experienced external rotation as PTS increased. In other words, internal rotation was reduced. However, there was no effect of the PTS in mobile-bearing rotating platform TKA. In the current study, the mobile-bearing rotating platform exhibited a contact point located near the mid-line of the tibia in extension. During a deep knee bend, mobile-bearing rotating platform TKA experienced an anterior slide of the femoral component, occurring at either 30° or 60° knee flexion. The mobile-bearing rotating platform TKA showed high conformity in 30° flexion, which explains the relatively neutral positioning in extension and flexion. Change in femoral geometry on the posterior condyles with a reduced radius of curvature led to the tendency toward anterior translation in deeper flexion.

In terms of clinical relevance, identifying the optimal PTS should help surgeons cut the proximal tibia properly on the sagittal alignment. However, there is no definition for optimal PTS. For fixed-bearing PS TKA, anterior impingement between the tibial post and the femoral component was observed at near-full extension in increased PTS [67]. In addition, increased PTS may lead to progressive loosening of the TF joint gap [36].

The results in this study showed that a thorough preplanning of the desired PTS should be performed before surgery, which considers the CR TKA design available and the surgical technique utilized, and that the execution of the tibial resections should be as precise as possible. The normal PTS in this study was about 6°, as measured from the available preoperative medical images. This native PTS may be an important parameter to consider in preoperative planning, which can also be easily measured from preoperative radiographs. We suggest that the surgeon preserves the patient's original PTS in CR TKA.

This study has some limitations. We performed the computational simulation using fixed- and mobile-bearing prostheses from different manufacturers. Therefore, the results from this experiment cannot be considered as representative of all fixed- and mobile-bearing TKA. Other types of fixed bearings or mobile bearings may provide different results. For instance, the J-curved prosthesis was used in this study, but the Scorpio (Stryker, Inc., Mahwah, NJ, USA) implant has femoral condyles that have a single medial-lateral (ML) radius of curvature, unlike many other CR implant designs that have separate centers of curvature for the medial and lateral condyles, in the coronal plane. Therefore, different prostheses and bearing types should be analyzed in the future. Further, although a deep-knee-bending simulation was performed, additional simulations related to more demanding activities (e.g., rising from a chair, sitting, and climbing and descending stairs) are required in the future for a more robust investigation. However, this simulation was performed for deep-knee-bending motions, because such motions include both a wide range of flexion and extension, and significant muscular effort around the knee joint. Another limitation is that the results cannot be utilized in place of clinical outcomes and do not consider patient satisfaction, because they correspond solely to the outcomes of computational analyses. However, the main factor analyzed in the present study corresponds to the main components investigated in evaluation of the biomechanical effect of computational biomechanics [23–25, 28, 40–43]. The computational model was developed using only data from a young and male subject. Using subjects of various ages would improve the validity of the results because the validity is also dependent upon the geometry of the knee joint. However, we overcome the problem of young model by comparison with previous experimental study. Finally, although the material properties and attachment points of the ligaments used in the model were assumed based on previously published studies, considerable variability exists. However, our objective was not to determine the quantitative values for muscle and ligament forces, but to determine the effects of variability in the PTS on our variables of interest.

In conclusion, the kinematics of fixed-bearing TKA changed as PTS changed, but there was relatively less change in mobile-bearing rotating platform TKA. In particular, for CR TKA, the effect of change in PTS was the greatest, which suggests that the surgeon should be careful in determining the PTS in CR TKA.

## Figures and Tables

**Table 1:** Material properties for FE model.

	Young's modulus (MPa)	Poisson's ratio
CoCrMo alloy	220,000	0.30
UHMWPE	685	0.47
Ti6Al4V alloy	110,000	0.30
PMMA	1,940	0.40

## Data Availability

All relevant data are within the paper.
